# Supplementary material for: Characteristics of non-drinking and drinking adults in Taiwan and their implications in alcohol epidemiological studies
Source: PLoS One. 2025 Apr 1;20(4):e0320451. doi: 10.1371/journal.pone.0320451 (PMC11960929; doi:10.1371/journal.pone.0320451)
Supplement: S1 Table — (DOCX) [file pone.0320451.s001.docx]

**Characteristics of Non-Drinking and Drinking Adults in Taiwan**

**and Their Implications in Alcohol Epidemiological Studies**

Tsung Yu, Tzu-Jung Wong and Hsing-Yu Yang

**Supplementary Materials**

**Table S1. Frequency of comorbidities in men (N = 2846) and women (N = 2833)**

| Comorbidities, n (%) | Men (N = 2846) | Women (N = 2833) |
| --- | --- | --- |
| Cataracts | 384 (13.5) | 499 (17.6) |
| Glaucoma | 63 (2.2) | 60 (2.1) |
| Tuberculosis | 39 (1.4) | 20 (0.7) |
| Emphysema | 11 (0.4) | 3 (0.1) |
| Chronic bronchitis | 71 (2.5) | 46 (1.6) |
| Peptic ulcer | 275 (9.7) | 247 (8.7) |
| Irritable bowel syndrome | 38 (1.3) | 29 (1.0) |
| Chronic hepatitis | 178 (6.3) | 96 (3.4) |
| Fatty liver disease | 309 (10.9) | 176 (6.2) |
| Cirrhosis | 37 (1.3) | 4 (0.1) |
| Thyroid disease | 59 (2.1) | 172 (6.1) |
| Gout | 308 (10.8) | 70 (2.5) |
| Arthritis | 114 (4.0) | 186 (6.6) |
| Hypertension | 758 (26.6) | 713 (25.2) |
| Hypercholesterolemia | 244 (8.6) | 210 (7.4) |
| Stroke | 87 (3.1) | 62 (2.2) |
| Diabetes mellitus | 322 (11.3) | 297 (10.5) |
| Chronic kidney disease | 59 (2.1) | 43 (1.5) |
| Kidney stone | 314 (11.0) | 190 (6.7) |
| Cardiovascular disease | 200 (7.0) | 199 (7.0) |
| Parkinson’s disease | 17 (0.6) | 17 (0.6) |
| Dementia | 1 (0.0) | 3 (0.1) |
| Depression | 41 (1.4) | 75 (2.7) |
| Anxiety | 34 (1.2) | 46 (1.6) |
| Other psychiatric disorders | 10 (0.4) | 11 (0.4) |
| Urinary incontinence | 19 (0.7) | 36 (1.3) |
| Benign prostatic hyperplasia | 318 (11.2) | - |
| Cancer | 77 (2.7) | 80 (2.8) |
