# Supplementary material for: Characteristics of non-drinking and drinking adults in Taiwan and their implications in alcohol epidemiological studies
Source: PLoS One. 2025 Apr 1;20(4):e0320451. doi: 10.1371/journal.pone.0320451 (PMC11960929; doi:10.1371/journal.pone.0320451)
Supplement: Attachment — Submitted filename: review comments_2.docx [file pone.0320451.s003.docx]

**Title: Characteristics of Non-Drinking and Drinking Adults in Taiwan and Their Implications in Alcohol Epidemiological Studies**

Thank you very much for allowing me to review this research article. The authors have addressed my comments and have incorporated them into the revised manuscript. I appreciate the time and effort the authors have towards manuscript revision. I highly recommended to accept this manuscript for publication.
